# Factors determining utilization of stem cell transplant for initial therapy of multiple myeloma by patient race: exploring intra-racial healthcare disparities

**DOI:** 10.1038/s41408-024-01067-x

**Published:** 2024-05-28

**Authors:** Sikander Ailawadhi, Yaw Adu, Ryan D. Frank, Saurav Das, David O. Hodge, Andre Fernandez, Caitlyn Flott, Jamie Elliott, Ricardo Parrondo, Taimur Sher, Vivek Roy, Asher A. Chanan-Khan

**Affiliations:** 1 Division of Hematology-Oncology, Mayo Clinic, Jacksonville, FL USA; 2 Texas Tech University Health Sciences Center School of Medicine, Lubbock, TX USA; 3 Division of Biomedical Statistics and Informatics, Mayo Clinic, Rochester, MN USA; 4 Department of Health Sciences Research/Biomedical Statistics and Informatics, Mayo Clinic, Jacksonville, FL USA

**Keywords:** Haematopoietic stem cells, Epidemiology, Cancer epidemiology, Myeloma, Quality of life

## Abstract

Multiple myeloma (MM) therapeutics have evolved tremendously in recent years, with significant improvement in patient outcomes. As newer treatment options are developed, stem cell transplant (SCT) remains an important modality that provides excellent disease control and delays the progression of disease. Over the years, SCT use has increased overall in the U.S., but two distinct gaps remain, including suboptimal use overall and racial-ethnic disparities. We evaluated the National Cancer Database (NCDB) to study what sociodemographic factors might play a role within a given racial-ethnic group leading to disparate SCT utilization, such that targeted approaches can be developed to optimize SCT use for all. In nearly 112,000 cases belonging to mutually exclusive categories of non-Hispanic Whites (NHW), non-Hispanic Blacks (NHB), Hispanics, non-Hispanic Asians (NHA), and others, we found certain factors including age, comorbidity index, payor type, facility type (academic vs. community) and facility volume to be uniformly associated with SCT use for all the racial-ethnic groups, while gender was not significant for any of the groups. There were several other factors that had a differential impact on SCT utilization among the various race-ethnicity groups studied, including year of diagnosis (significant for NHW, NHB, and Hispanics), income level (significant for NHW and Hispanics), literacy level (significant for NHW and NHB), and geographic location of the treatment facility (significant for NHW and NHA). The suboptimal SCT utilization overall in the U.S. suggests that there may be room for improvement for all, even including the majority NHW, while we continue to work on factors that lead to disparities for the traditionally underserved populations. This study helps identify sociodemographic factors that may play a role specifically in each group and paves the way to devise targeted solutions such that resource utilization and impact can be maximized.

## Introduction

Multiple myeloma (MM) is the second most common hematologic malignancy in the United States (US), with an increasing incidence noted over time [1]. Risk factors for MM include age, race, gender, and family history, while older age and certain chromosomal abnormalities have been identified as adverse prognostic factors [2]. MM remains an incurable disorder, however, over the past two decades therapeutic advancements, as well as increasing utilization of modalities such as stem cell transplant (SCT), has led to improving patient outcomes [1, 3–5]. The population-wide estimated 5-year overall survival (OS) has improved over time, with the most recent data showing 55% [6].

Depending on age and risk stratification, the current treatment for all eligible patients with newly diagnosed MM involves induction therapy coupled with SCT, followed by a maintenance regimen aimed at delaying disease recurrence [1, 5]. SCT has been the standard of care for MM patients considered eligible as per clinical assessment, as it has been associated with a definitive improvement in progression-free survival (PFS) [5]. Despite this proven benefit, there is evidence that only a small proportion of patients who are considered transplant-eligible receive SCT in the US [7–9]. Currently, there is evidence supporting the existence of racial-ethnic disparities in treatment outcomes and management of patients with MM [8–13]. Historically, studies assessing racial disparities primarily focused on comparisons between African Americans and Whites, despite the notion that this approach may not accurately reflect the evolving demographics of the United States. Notably, the population of Hispanics and Asians has steadily grown in the US, but they have had limited or no representation in MM outcomes research [14, 15].

Furthermore, while overall SCT utilization has been increasing over time in the US, there is a large body of evidence highlighting disparities, where patients who belong to racial-ethnic minorities have a significantly lesser and delayed SCT utilization as compared to the predominant whites [9, 11, 12]. Studies reporting such significant differences in induction therapies, access to care, and SCT utilization uncover the exacerbating disparity in survival rates among racial-ethnic groups [9, 11, 12, 16]. Different studies have shown various sociodemographic factors that may be associated with disparate SCT utilization, yet there is a varying proportion of patients in each racial group that do undergo SCT as part of MM management. Factors that may be in play within a racial-ethnic group associated with the likelihood of receiving SCT for MM have not been previously explored. Understanding such factors may help in formulating targeted strategies that are applicable to a given racial-ethnic group such that optimal SCT utilization can be achieved across the racial-ethnic distribution.

## Subjects and methods

### Data source and patient selection

We conducted a retrospective analysis using de-identified data accessed from the NCDB, which has previously been reported for such analyses [17]. The study was exempt from Institutional Review Board (IRB) oversight and did not require Ethics approval.

NCDB was queried for patients diagnosed between 2004 and 2013 with MM (ICD-0-3 code 9732) who had undertaken SCT as initial therapy. NCDB does not identify patients as active myeloma or smoldering myeloma at diagnosis. Therefore, we excluded patients who did not receive any therapy for the diagnosis of MM and included only those who were treated within 120 days of MM diagnosis based on a methodology similar to that utilized in previous studies [18]. The “Class of case” variable in NCDB determines whether diagnosis and treatment of the disease were conducted at the same facility or not. We excluded patients with the class of case categories other than 10-14 (patients who had a diagnosis at the reporting facility and all treatment or a decision not to treat was performed elsewhere), as has been previously utilized and reported from the NCDB, to minimize reporting errors [17]. Race-ethnicity was described into mutually exclusive categories: non-Hispanic Whites (NHW), non-Hispanic Blacks (NHB), Hispanics, non-Hispanic Asians (NHA), and others. Patient characteristics provided in NCDB were utilized for the analysis, including sociodemographic characteristics such as age, gender, year of diagnosis, median household income, education level (quartiles of the percentage of persons with less than a high school education), distance from treating facility, Charlson-comorbidity index (CCI), insurance status (captured in NCDB as it appears on the admission face sheet for the patient and was recorded as insured; Private, Medicaid, Medicare, others, or uninsured), treatment options such as the use of SCT as initial therapy, and geographic region (East coast, Central, Mountain, and Pacific). Facility factors indicated whether the type of facility care was at an academic or a community center according to the Commission on Cancer (CoC) accreditation category as used in the NCDB.

### Statistical analysis

Patient factors and SCT utilization were described using frequencies or percentages for categorical variables and median or interquartile ranges for continuous variables. Formal comparisons across race and the factors associated with SCT utilization were determined using a multivariable logistic regression model after adjusting for age, gender, year of diagnosis, median household income, education level, distance from treating facility, CCI, insurance status, treating facility type, and geographic region. Patients with missing data were not included in analyses for that particular covariate, and data was not imputed. Associations between SCT utilization and sociodemographic factors for each racial group clustered on facility type were summarized separately for each race using a proportional odds model. SCT use by race interaction was analyzed using similar methods. All analyses were performed using SAS version 9.4 (SAS Institute, Inc., Cary, NC), with *P* < 0.05 indicating significance.

## Results

**Fig. 1:**
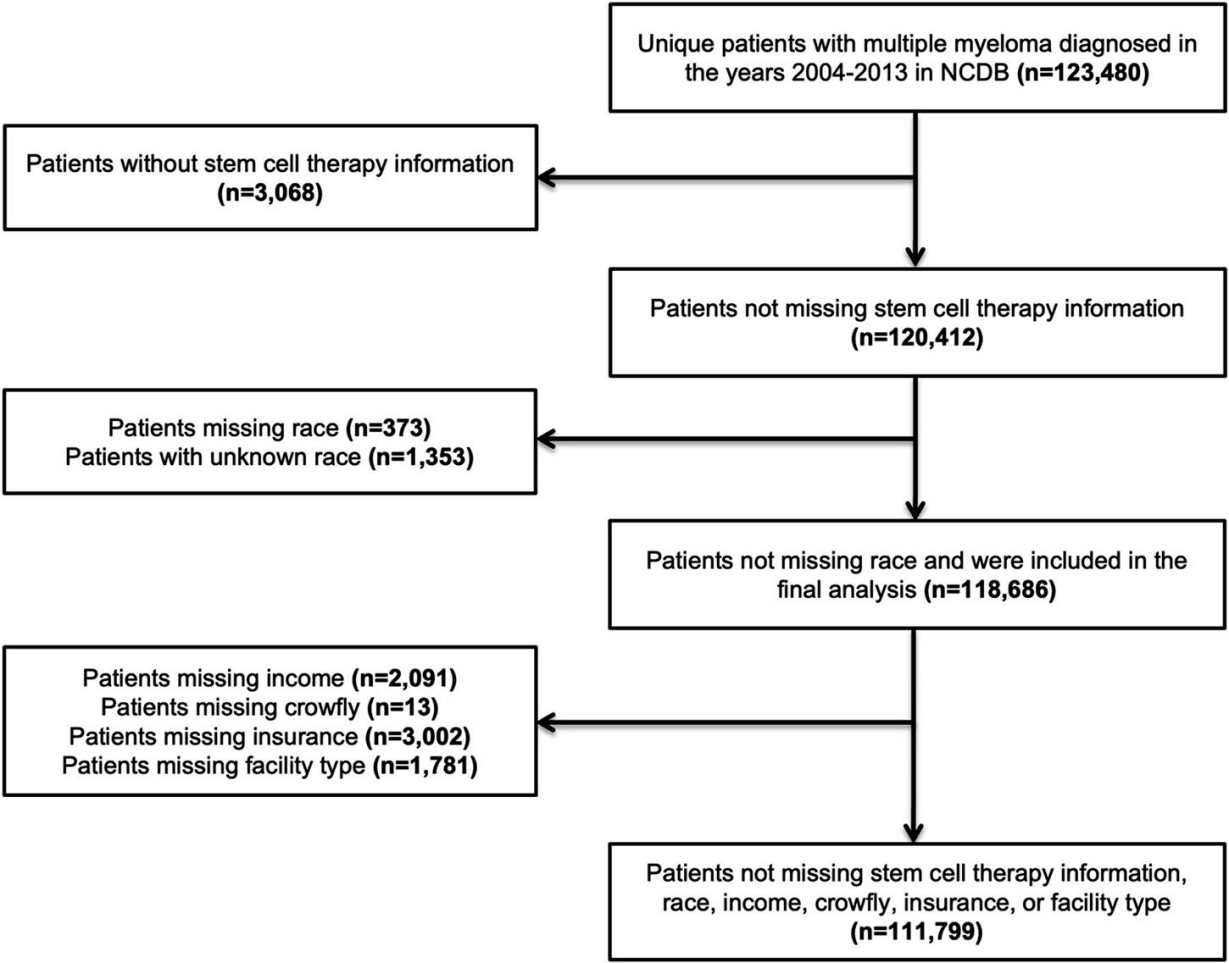
Consort diagram showing exclusion criteria leading to final cohort for analysis.

From 2003 to 2014, a total of 123,480 MM patient records with unique IDs were identified in the NCDB database, of which 3,068 records without any information on SCT use and 1726 records with missing or unknown information on race were removed. Further, 6887 records with missing information on median income, insurance status, and facility type were removed. In total, 111,799 patient records diagnosed with MM in this time frame with all required information were included in the final analysis (Fig. 1). Of those who received SCT, race-ethnicity category distribution showed that 77.5% were NHW (14.4% of all NHW), 15.1% NHB (10.3% of all NHB), 5.2% Hispanic (13.2% of all Hispanic), 2% NHA (15.5% of all NHA) and <1% were others.

The odds of receiving SCT were significantly decreased for every 10-year increase in age at diagnosis for all racial-ethnic subgroups. There was no significant association between gender and SCT utilization among any of the racial-ethnic groups. For all races except NHA, the odds of SCT utilization significantly increased over time year-wise between 2004 and 2013. Certain socioeconomic factors were associated with significantly increased odds of SCT utilization, including an increase in median income (for NHW and Hispanic, but not for NHB or NHA) and higher education (for NHW and NHB, but not for Hispanic or NHA). An increase in great circle distance (GCD) from the treating facility and a lower CCI was associated with significantly increased odds of receiving SCT for all racial-ethnic groups. Insurance payer status significantly affected the odds of receiving SCT among all races. Those with private insurance were the most likely to receive SCT among NHW, NHB, and Hispanics, while those with “other government” insurance payer type were most likely among NHA. Characteristics of the treating facility were uniformly associated with the odds of receiving SCT among all race-ethnicity groups. Patients in all groups were more likely to receive SCT when treated at an academic or research facility, including National Cancer Institute (NCI) designated centers or centers with the highest quartile of patient volume seen. US geographical regions did not affect the odds of receiving SCT among NHB or Hispanics while both NHW and NHA were more likely to receive SCT in the Mountain region as compared to other geographical distributions. The interaction between the odds of receiving SCT and various racial subgroups is presented in Table 1. Details of the association between the odds of receiving SCT as part of initial MM therapy and the various sociodemographic factors studied separately for each of the racial-ethnic groups are given in Supplementary Tables S1a–d.

**Table 1:** Associations of patient characteristics with odds of receiving stem cell transplant by race-ethnicity.

Covariate	NHW	NWB	Hispanic	NHA	Other	p-value†
	OR (95% CI)	OR (95% CI)	OR (95% CI)	OR (95% CI)	OR (95% CI)	
*Patient demographics*						
Age (per 10 year increase)	0.46 (0.44, 0.48)***	0.51 (0.48, 0.54)***	0.55 (0.50, 0.61)***	0.45 (0.38, 0.54)***	0.49 (0.39, 0.62)***	<0.001
*Year of diagnosis (ref 2004, 2005)*						0.630
2006–2007	1.09 (0.98, 1.21)	1.02 (0.82, 1.26)	1.00 (0.67, 1.50)	0.99 (0.51, 1.91)	0.73 (0.25, 2.10)	
2008–2009	1.36 (1.20, 1.55)***	1.43 (1.19, 1.73)***	1.48 (1.04, 2.10)*	0.87 (0.50, 1.54)	1.16 (0.46, 2.96)	
2010–2011	1.91 (1.69, 2.16)***	1.71 (1.42, 2.07)***	1.83 (1.24, 2.70)**	1.34 (0.81, 2.23)	1.74 (0.73, 4.16)	
2012–2013	1.97 (1.72, 2.25)***	2.04 (1.64, 2.55)***	2.08 (1.44, 2.99)***	1.30 (0.76, 2.24)	2.08 (0.87, 4.99)	
*Median income (ref <$38k)*						0.324
$38,000–$47,999	1.10 (0.99, 1.23)	1.17 (0.98, 1.39)	1.34 (1.01, 1.78)*	2.20 (0.84, 5.73)	1.09 (0.57, 2.10)	
$48,000–$62,999	1.19 (1.04, 1.37)*	1.28 (1.03, 1.60)*	1.62 (1.19, 2.21)**	2.81 (1.07, 7.39)*	1.72 (0.83, 3.53)	
$63,000+	1.13 (0.90, 1.40)	1.28 (0.96, 1.69)	2.09 (1.39, 3.14)***	2.82 (1.01, 7.90)*	1.21 (0.54, 2.71)	
*% No HSD (ref ≥21%)*						0.257
13–20%	1.21 (1.06, 1.37)**	1.09 (0.94, 1.26)	0.95 (0.70, 1.29)	0.83 (0.44, 1.55)	0.70 (0.37, 1.34)	
7.0–12.9%	1.40 (1.17, 1.67)***	1.24 (1.00, 1.54)*	0.89 (0.62, 1.28)	0.92 (0.47, 1.79)	1.08 (0.57, 2.04)	
<7%	1.55 (1.25, 1.92)***	1.51 (1.15, 1.98)**	0.77 (0.46, 1.27)	0.79 (0.39, 1.60)	0.93 (0.44, 1.98)	
GCD (per 10-mile Increase)	1.10 (1.07, 1.13)***	1.14 (1.11, 1.17)***	1.10 (1.07, 1.13)***	1.15 (1.11, 1.20)***	1.11 (1.07, 1.15)***	0.013
*Charlson-Deyo Score (ref 0)*						0.043
1	0.83 (0.76, 0.90)***	0.98 (0.82, 1.17)	0.92 (0.72, 1.18)	0.86 (0.54, 1.36)	1.56 (0.85, 2.85)	
2+	0.47 (0.41, 0.54)***	0.46 (0.35, 0.60)***	0.50 (0.31, 0.81)**	0.31 (0.11, 0.87)*	1.35 (0.61, 2.98)	
*Primary Payor (ref not insured)*						0.115
Private Insurance	5.22 (4.08, 6.68)***	5.00 (3.46, 7.25)***	9.51 (4.09, 22.11)***	4.35 (1.65, 11.49)**	24.70 (3.32, 183.87)**	
Medicaid	2.62 (2.00, 3.42)***	2.37 (1.68, 3.34)***	4.11 (1.88, 8.97)***	1.38 (0.50, 3.82)	11.26 (1.42, 89.01)*	
Medicare	3.60 (2.83, 4.59)***	3.04 (2.11, 4.37)***	4.68 (2.11, 10.35)***	2.22 (0.82, 5.96)	17.16 (3.23, 91.23)***	
Other Government	3.85 (2.76, 5.36)***	3.77 (2.12, 6.70)***	4.58 (1.65, 12.71)**	8.74 (1.83, 41.62)**	15.22 (2.13, 108.77)**	
*Facility characteristics*						
Academic/Research Program	2.91 (2.24, 3.79)***	2.70 (1.88, 3.89)***	2.83 (1.73, 4.63)***	2.89 (1.55, 5.38)***	2.79 (1.47, 5.29)**	0.989
*Patients/year quartiles (ref Quartile 1)*						0.046
Quartile 2	1.06 (0.79, 1.42)	1.44 (0.67, 3.10)	1.35 (0.41, 4.49)	0.07 (0.00, 1.04)	1.00 (0.10, 10.43)	
Quartile 3	0.97 (0.72, 1.30)	1.58 (0.74, 3.36)	1.39 (0.43, 4.48)	1.04 (0.29, 3.82)	1.29 (0.14, 11.93)	
Quartile 4	2.34 (1.70, 3.21)***	4.87 (2.30, 10.32)***	6.05 (1.91, 19.17)**	3.88 (1.16, 12.97)*	2.71 (0.36, 20.28)	

*NWH* non-Hispanic White, *NWB* non-Hispanic Black, *NHA* non-Hispanic Asian, *OR* odds ratio, *HSD* high school degree, *GCD* great circle distance.

*P < 0.05 **P < 0.01 ***P < 0.001.

^†^Race by covariate interaction *P*-value. *P* < 0.05 indicates that the association with SCT was significantly different by racial category.

Other than showing the above-mentioned associations between the various covariates and odds of receiving SCT, the multivariate analysis also showed that age was more strongly associated with increased SCT utilization among NHW and NHA than NHB and Hispanics, whereas GCD was more strongly associated with NHB and NHA than NHW and Hispanics. Likewise, the CCI score was a strong protective association for all minority races but had no association among “other” races. Facility volume (myeloma diagnoses per year) had weaker associations among NHW and “others” than NHB, Hispanics, and NHA (Table 1).

## Discussion

Health equity and uniform opportunities for all are imperative to achieve anticipated evidence-based medical outcomes. Yet, parity in access and utilization of healthcare resources have been shown to be significantly disparate among patients belonging to different racial-ethnic and socioeconomic groups in the population at-large. There is increasing awareness of this disparity among healthcare providers, patients, advocacy groups, and policymakers. Frequently, racial-ethnic disparities have been explored as differences amid the predominant Whites and minority NHB, with occasional studies including Hispanics and NHA as well [9, 12, 15, 16, 19–22]. While such disparities are prevalent and must be addressed, we hypothesized that the factors contributing to them might be unique for a given racial-ethnic group, and as a result, the solutions needed to address them may have to be designed correspondingly. We took a unique approach to explore intra-racial disparities among patients who do or do not receive SCT as part of initial therapy for MM.

In our analysis, NHB was the racial-ethnic group with the lowest proportion of patients who received SCT for MM. This was not a formal, adjusted analysis, but previous studies have also shown that racial-ethnic minorities tend to get SCT less frequently as compared to Whites [9, 12]. We noted that NHA was the only group that did not have a significant increase in SCT utilization over time. Previous reports that mentioned an increase in SCT use for all racial-ethnic groups did not include NHA and focused only on Whites, NHB, and Hispanics [9]. Age and performance status are important considerations in determining a patient’s eligibility for SCT [10, 23]. In our analysis, as expected, both increasing age and CCI were associated with lower odds of receiving SCT for all racial-ethnic groups, consistent with standard clinical practice. Novel anti-MM agents are making it possible to achieve better disease control and perform SCT even in patients with older age and higher disease burden, leading to superior outcomes [4, 11, 19, 24]. We have previously shown that access to novel therapeutic agents may be delayed and lower among racial-ethnic minorities, which can lead to poor disease control and, in turn, reduced eligibility for SCT [11, 12].

While the incidence of MM is different for men and women, we did not note an association of gender with the odds of receiving SCT for any of the racial-ethnic groups [6]. An increase in GCD from the treating facility was associated with higher odds of receiving SCT for all racial-ethnic groups. We hypothesize that patients who were already traveling a longer distance to receive their MM treatment had the means, access, and support system that facilitated receiving a relatively resource-intensive treatment modality such as SCT. Private insurance status was more likely to be associated with getting SCT among NHW, NHB, and Hispanics. Having private insurance is associated with a higher socioeconomic status (SES), being employed, and having resources at disposal, which can help facilitate a rather resource-intensive SCT process [25, 26]. Receiving care at large volumes and academic medical centers has been previously reported to be associated with superior outcomes in MM care [27, 28]. SCT access and utilization could certainly play a role in this, among other factors such as access to clinical trials and evidence-based care in general.

Healthcare disparities in MM have been primarily considered due to differences in access to care, frequently a result of socioeconomic disadvantage [12, 17]. Studies have shown how racial-ethnic minorities, including NHB and Hispanics, are at a disadvantage in accessing and receiving SCT [12, 14, 16]. It is also reported that the overall SCT utilization rates are relatively low in the U.S., suggesting that SCT use may not be optimal even for the racial-ethnic majority, in addition to the traditionally underserved groups [29]. We report what socioeconomic factors might be playing a role within a specific racial-ethnic group. This provides an opportunity to target awareness and intervention campaigns that are tailored to the needs of the specific group such that awareness and utilization of SCT are optimized for all.

### Supplementary information

Supplementary Tables

## Data Availability

Study data is available on request from NCI as per the NCDB data guidelines and processes (National Cancer Database (NCDB) | ACS (facs.org)).
