# Supplementary material for: Factors determining utilization of stem cell transplant for initial therapy of multiple myeloma by patient race: exploring intra-racial healthcare disparities
Source: Blood Cancer J. 2024 May 28;14(1):86. doi: 10.1038/s41408-024-01067-x (PMC11133435; doi:10.1038/s41408-024-01067-x)
Supplement: Supplementary file 1 — Supplementary Tables [file 41408_2024_1067_MOESM1_ESM.docx]

**Supplementary Table S1a.** **Associations of Patient Characteristics in Stem Cell Transplant Patients for Non-Hispanic Whites (NHW)**

| Covariate | Total  N=80765 | N; event | Odds Ratio  (95% CI) | p-value | p-value for covariate |
| --- | --- | --- | --- | --- | --- |
| Age per 10 Year Increase |  |  |  |  | <.001 |
| *Ordinal Effect* | 80765 | 11644 (14.4%) | 0.46 (0.44, 0.48) | <.001 |  |
| Year of Diagnosis |  |  |  |  | <.001 |
| *2004-2005* | 13186 | 1339 (10.2%) | 1.00 (ref) | - |  |
| *2006-2007* | 14360 | 1635 (11.4%) | 1.09 (0.98, 1.21) | 0.120 |  |
| *2008-2009* | 15936 | 2177 (13.7%) | 1.36 (1.20, 1.55) | <.001 |  |
| *2010-2011* | 17822 | 3079 (17.3%) | 1.91 (1.69, 2.16) | <.001 |  |
| *2012-2013* | 19461 | 3414 (17.5%) | 1.97 (1.72, 2.25) | <.001 |  |
| Sex |  |  |  |  | 0.109 |
| *Female* | 35010 | 4784 (13.7%) | 1.00 (ref) | - |  |
| *Male* | 45755 | 6860 (15.0%) | 1.04 (0.99, 1.08) | 0.109 |  |
| Median Income Quartiles 2008-2012 |  |  |  |  | 0.016 |
| *<$38,000* | 10889 | 1243 (11.4%) | 1.00 (ref) | - |  |
| *$38,000-$47,999* | 19497 | 2546 (13.1%) | 1.10 (0.99, 1.23) | 0.086 |  |
| *$48,000-$62,999* | 22683 | 3329 (14.7%) | 1.19 (1.04, 1.37) | 0.013 |  |
| *$63,000 +* | 27696 | 4526 (16.3%) | 1.13 (0.90, 1.40) | 0.288 |  |
| Percent No High School Degree 2008-2012 |  |  |  |  | 0.001 |
| *>=21%* | 9406 | 1007 (10.7%) |  |  |  |
| *13-20%* | 19464 | 2461 (12.6%) | 1.21 (1.06, 1.37) | 0.004 |  |
| *7.0-12.9%* | 28663 | 4160 (14.5%) | 1.40 (1.17, 1.67) | <.001 |  |
| *<7%* | 23232 | 4016 (17.3%) | 1.55 (1.25, 1.92) | <.001 |  |
| Great Circle Distance per 10 Unit Increase |  |  |  |  | <.001 |
| *Ordinal Effect* | 80765 | 11644 (14.4%) | 1.10 (1.07, 1.13) | <.001 |  |
| Charlson-Deyo Comorbidity Index Score |  |  |  |  | <.001 |
| *0* | 62401 | 10054 (16.1%) | 1.00 (ref) | - |  |
| *1* | 12497 | 1284 (10.3%) | 0.83 (0.76, 0.90) | <.001 |  |
| *2+* | 5867 | 306 (5.2%) | 0.47 (0.41, 0.54) | <.001 |  |
| Primary Payor |  |  |  |  | <.001 |
| *Not Insured* | 1591 | 115 (7.2%) | 1.00 (ref) | - |  |
| *Private Insurance* | 29346 | 7938 (27.0%) | 5.22 (4.08, 6.68) | <.001 |  |
| *Medicaid* | 2264 | 387 (17.1%) | 2.62 (2.00, 3.42) | <.001 |  |
| *Medicare* | 46774 | 3050 (6.5%) | 3.60 (2.83, 4.59) | <.001 |  |
| *Other Government* | 790 | 154 (19.5%) | 3.85 (2.76, 5.36) | <.001 |  |
|  |  |  |  |  |  |
| **Facility Characteristics** |  |  |  |  |  |
| Academic/Research Program Facility Type | 29654 | 8454 (28.5%) | 2.91 (2.24, 3.79) | <.001 | <.001 |
| Geographic region |  |  |  |  | 0.002 |
| *East Coast* | 31995 | 4067 (12.7%) | 1.00 (ref) | - |  |
| *Central* | 35415 | 5359 (15.1%) | 1.49 (1.16, 1.93) | 0.002 |  |
| *Mountain* | 4500 | 953 (21.2%) | 2.16 (1.22, 3.81) | 0.008 |  |
| *Pacific* | 8855 | 1265 (14.3%) | 1.47 (0.96, 2.27) | 0.077 |  |
| Patients/year quartiles |  |  |  |  | <.001 |
| *Q1* | 5414 | 194 (3.6%) | 1.00 (ref) | - |  |
| *Q2* | 10379 | 454 (4.4%) | 1.06 (0.79, 1.42) | 0.711 |  |
| *Q3* | 17963 | 853 (4.7%) | 0.97 (0.72, 1.30) | 0.822 |  |
| *Q4* | 47009 | 10143 (21.6%) | 2.34 (1.70, 3.21) | <.001 |  |

**Supplementary Table S1b.** **Associations of Patient Characteristics in Stem Cell Transplant Patients for Non-Hispanic Blacks (NHB)**

| Covariate | Total  N=22113 | N; event | Odds Ratio  (95% CI) | p-value | p-value for covariate |
| --- | --- | --- | --- | --- | --- |
| Age per 10 Year Increase |  |  |  |  | <.001 |
| *Ordinal Effect* | 22113 | 2275 (10.3%) | 0.51 (0.48, 0.54) | <.001 |  |
| Year of Diagnosis |  |  |  |  | <.001 |
| *2004-2005* | 3310 | 234 (7.1%) | 1.00 (ref) | - |  |
| *2006-2007* | 3662 | 265 (7.2%) | 1.02 (0.82, 1.26) | 0.852 |  |
| *2008-2009* | 4418 | 437 (9.9%) | 1.43 (1.19, 1.73) | <.001 |  |
| *2010-2011* | 5065 | 600 (11.8%) | 1.71 (1.42, 2.07) | <.001 |  |
| *2012-2013* | 5658 | 739 (13.1%) | 2.04 (1.64, 2.55) | <.001 |  |
| Sex |  |  |  |  | 0.476 |
| *Female* | 11511 | 1166 (10.1%) | 1.00 (ref) | - |  |
| *Male* | 10602 | 1109 (10.5%) | 0.96 (0.86, 1.07) | 0.476 |  |
| Median Income Quartiles 2008-2012 |  |  |  |  | 0.160 |
| *<$38,000* | 9521 | 765 (8.0%) | 1.00 (ref) | - |  |
| *$38,000-$47,999* | 4997 | 507 (10.1%) | 1.17 (0.98, 1.39) | 0.082 |  |
| *$48,000-$62,999* | 4239 | 519 (12.2%) | 1.28 (1.03, 1.60) | 0.028 |  |
| *$63,000 +* | 3356 | 484 (14.4%) | 1.28 (0.96, 1.69) | 0.090 |  |
| Percent No High School Degree 2008-2012 |  |  |  |  | 0.029 |
| *>=21%* | 7608 | 613 (8.1%) |  |  |  |
| *13-20%* | 8067 | 780 (9.7%) | 1.09 (0.94, 1.26) | 0.268 |  |
| *7.0-12.9%* | 4498 | 553 (12.3%) | 1.24 (1.00, 1.54) | 0.047 |  |
| *<7%* | 1940 | 329 (17.0%) | 1.51 (1.15, 1.98) | 0.003 |  |
| Great Circle Distance per 10 Unit Increase |  |  |  |  | <.001 |
| *Ordinal Effect* | 22113 | 2275 (10.3%) | 1.14 (1.11, 1.17) | <.001 |  |
| Charlson-Deyo Comorbidity Index Score |  |  |  |  | <.001 |
| *0* | 15826 | 1825 (11.5%) | 1.00 (ref) | - |  |
| *1* | 4110 | 358 (8.7%) | 0.98 (0.82, 1.17) | 0.842 |  |
| *2+* | 2177 | 92 (4.2%) | 0.46 (0.35, 0.60) | <.001 |  |
| Primary Payor |  |  |  |  | <.001 |
| *Not Insured* | 1289 | 66 (5.1%) | 1.00 (ref) | - |  |
| *Private Insurance* | 7565 | 1422 (18.8%) | 5.00 (3.46, 7.25) | <.001 |  |
| *Medicaid* | 2246 | 226 (10.1%) | 2.37 (1.68, 3.34) | <.001 |  |
| *Medicare* | 10721 | 514 (4.8%) | 3.04 (2.11, 4.37) | <.001 |  |
| *Other Government* | 292 | 47 (16.1%) | 3.77 (2.12, 6.70) | <.001 |  |
|  |  |  |  |  |  |
| **Facility Characteristics** |  |  |  |  |  |
| Academic/Research Program Facility Type | 10828 | 1783 (16.5%) | 2.70 (1.88, 3.89) | <.001 | <.001 |
| Geographic region |  |  |  |  | 0.056 |
| *East Coast* | 11639 | 1106 (9.5%) | 1.00 (ref) | - |  |
| *Central* | 9056 | 947 (10.5%) | 1.45 (1.05, 2.01) | 0.024 |  |
| *Mountain* | 251 | 53 (21.1%) | 2.48 (0.96, 6.37) | 0.059 |  |
| *Pacific* | 1167 | 169 (14.5%) | 1.69 (0.68, 4.20) | 0.260 |  |
| Patients/year quartiles |  |  |  |  | <.001 |
| *Q1* | 816 | 12 (1.5%) | 1.00 (ref) | - |  |
| *Q2* | 2111 | 55 (2.6%) | 1.44 (0.67, 3.10) | 0.356 |  |
| *Q3* | 4190 | 127 (3.0%) | 1.58 (0.74, 3.36) | 0.239 |  |
| *Q4* | 14996 | 2081 (13.9%) | 4.87 (2.30, 10.32) | <.001 |  |

**Supplementary Table S1c.** **Associations of Patient Characteristics in Stem Cell Transplant Patients for Hispanics**

| Covariate | Total  N=5962 | N; event | Odds Ratio  (95% CI) | p-value | p-value for covariate |
| --- | --- | --- | --- | --- | --- |
| Age per 10 Year Increase |  |  |  |  | <.001 |
| *Ordinal Effect* | 5962 | 789 (13.2%) | 0.55 (0.50, 0.61) | <.001 |  |
| Year of Diagnosis |  |  |  |  | <.001 |
| *2004-2005* | 861 | 80 (9.3%) | 1.00 (ref) | - |  |
| *2006-2007* | 945 | 89 (9.4%) | 1.00 (0.67, 1.50) | 0.997 |  |
| *2008-2009* | 1199 | 149 (12.4%) | 1.48 (1.04, 2.10) | 0.029 |  |
| *2010-2011* | 1382 | 218 (15.8%) | 1.83 (1.24, 2.70) | 0.002 |  |
| *2012-2013* | 1575 | 253 (16.1%) | 2.08 (1.44, 2.99) | <.001 |  |
| Sex |  |  |  |  | 0.613 |
| *Female* | 2640 | 343 (13.0%) | 1.00 (ref) | - |  |
| *Male* | 3322 | 446 (13.4%) | 0.96 (0.83, 1.12) | 0.613 |  |
| Median Income Quartiles 2008-2012 |  |  |  |  | 0.004 |
| *<$38,000* | 1589 | 149 (9.4%) | 1.00 (ref) | - |  |
| *$38,000-$47,999* | 1487 | 188 (12.6%) | 1.34 (1.01, 1.78) | 0.043 |  |
| *$48,000-$62,999* | 1621 | 229 (14.1%) | 1.62 (1.19, 2.21) | 0.002 |  |
| *$63,000 +* | 1265 | 223 (17.6%) | 2.09 (1.39, 3.14) | <.001 |  |
| Percent No High School Degree 2008-2012 |  |  |  |  | 0.733 |
| *>=21%* | 3049 | 346 (11.3%) |  |  |  |
| *13-20%* | 1342 | 188 (14.0%) | 0.95 (0.70, 1.29) | 0.751 |  |
| *7.0-12.9%* | 1014 | 163 (16.1%) | 0.89 (0.62, 1.28) | 0.532 |  |
| *<7%* | 557 | 92 (16.5%) | 0.77 (0.46, 1.27) | 0.306 |  |
| Great Circle Distance per 10 Unit Increase |  |  |  |  | <.001 |
| *Ordinal Effect* | 5962 | 789 (13.2%) | 1.10 (1.07, 1.13) | <.001 |  |
| Charlson-Deyo Comorbidity Index Score |  |  |  |  | 0.018 |
| *0* | 4469 | 654 (14.6%) | 1.00 (ref) | - |  |
| *1* | 1044 | 110 (10.5%) | 0.92 (0.72, 1.18) | 0.515 |  |
| *2+* | 449 | 25 (5.6%) | 0.50 (0.31, 0.81) | 0.005 |  |
| Primary Payor |  |  |  |  | <.001 |
| *Not Insured* | 652 | 26 (4.0%) | 1.00 (ref) | - |  |
| *Private Insurance* | 1959 | 485 (24.8%) | 9.51 (4.09, 22.11) | <.001 |  |
| *Medicaid* | 927 | 126 (13.6%) | 4.11 (1.88, 8.97) | <.001 |  |
| *Medicare* | 2372 | 144 (6.1%) | 4.68 (2.11, 10.35) | <.001 |  |
| *Other Government* | 52 | 8 (15.4%) | 4.58 (1.65, 12.71) | 0.003 |  |
|  |  |  |  |  |  |
| **Facility Characteristics** |  |  |  |  |  |
| Academic/Research Program Facility Type | 2658 | 595 (22.4%) | 2.83 (1.73, 4.63) | <.001 | <.001 |
| Geographic region |  |  |  |  | 0.271 |
| *East Coast* | 2054 | 238 (11.6%) | 1.00 (ref) | - |  |
| *Central* | 1729 | 201 (11.6%) | 1.40 (0.86, 2.27) | 0.180 |  |
| *Mountain* | 478 | 95 (19.9%) | 2.05 (0.93, 4.54) | 0.077 |  |
| *Pacific* | 1701 | 255 (15.0%) | 1.20 (0.49, 2.91) | 0.692 |  |
| Patients/year quartiles |  |  |  |  | <.001 |
| *Q1* | 232 | 4 (1.7%) | 1.00 (ref) | - |  |
| *Q2* | 749 | 21 (2.8%) | 1.35 (0.41, 4.49) | 0.622 |  |
| *Q3* | 1242 | 46 (3.7%) | 1.39 (0.43, 4.48) | 0.578 |  |
| *Q4* | 3739 | 718 (19.2%) | 6.05 (1.91, 19.17) | 0.002 |  |

**Supplementary Table S1d.** **Associations of Patient Characteristics in Stem Cell Transplant Patients for Non-Hispanic Asians (NHA)**

| Covariate | Total  N=2016 | N; event | Odds Ratio  (95% CI) | p-value | p-value for covariate |
| --- | --- | --- | --- | --- | --- |
| Age per 10 Year Increase |  |  |  |  | <.001 |
| *Ordinal Effect* | 2016 | 313 (15.5%) | 0.45 (0.38, 0.54) | <.001 |  |
| Year of Diagnosis |  |  |  |  | 0.277 |
| *2004-2005* | 273 | 35 (12.8%) | 1.00 (ref) | - |  |
| *2006-2007* | 330 | 47 (14.2%) | 0.99 (0.51, 1.91) | 0.978 |  |
| *2008-2009* | 362 | 50 (13.8%) | 0.87 (0.50, 1.54) | 0.642 |  |
| *2010-2011* | 497 | 90 (18.1%) | 1.34 (0.81, 2.23) | 0.253 |  |
| *2012-2013* | 554 | 91 (16.4%) | 1.30 (0.76, 2.24) | 0.339 |  |
| Sex |  |  |  |  | 0.258 |
| *Female* | 910 | 130 (14.3%) | 1.00 (ref) | - |  |
| *Male* | 1106 | 183 (16.5%) | 1.17 (0.89, 1.54) | 0.258 |  |
| Median Income Quartiles 2008-2012 |  |  |  |  | 0.216 |
| *<$38,000* | 148 | 11 (7.4%) | 1.00 (ref) | - |  |
| *$38,000-$47,999* | 268 | 36 (13.4%) | 2.20 (0.84, 5.73) | 0.107 |  |
| *$48,000-$62,999* | 504 | 86 (17.1%) | 2.81 (1.07, 7.39) | 0.036 |  |
| *$63,000 +* | 1096 | 180 (16.4%) | 2.82 (1.01, 7.90) | 0.049 |  |
| Percent No High School Degree 2008-2012 |  |  |  |  | 0.842 |
| *>=21%* | 422 | 48 (11.4%) |  |  |  |
| *13-20%* | 409 | 53 (13.0%) | 0.83 (0.44, 1.55) | 0.557 |  |
| *7.0-12.9%* | 628 | 108 (17.2%) | 0.92 (0.47, 1.79) | 0.802 |  |
| *<7%* | 557 | 104 (18.7%) | 0.79 (0.39, 1.60) | 0.505 |  |
| Great Circle Distance per 10 Unit Increase |  |  |  |  | <.001 |
| *Ordinal Effect* | 2016 | 313 (15.5%) | 1.15 (1.11, 1.20) | <.001 |  |
| Charlson-Deyo Comorbidity Index Score |  |  |  |  | 0.046 |
| *0* | 1576 | 276 (17.5%) | 1.00 (ref) | - |  |
| *1* | 312 | 33 (10.6%) | 0.86 (0.54, 1.36) | 0.508 |  |
| *2+* | 128 | 4 (3.1%) | 0.31 (0.11, 0.87) | 0.026 |  |
| Primary Payor |  |  |  |  | <.001 |
| *Not Insured* | 107 | 10 (9.3%) | 1.00 (ref) | - |  |
| *Private Insurance* | 810 | 230 (28.4%) | 4.35 (1.65, 11.49) | 0.003 |  |
| *Medicaid* | 241 | 22 (9.1%) | 1.38 (0.50, 3.82) | 0.535 |  |
| *Medicare* | 838 | 44 (5.3%) | 2.22 (0.82, 5.96) | 0.115 |  |
| *Other Government* | 20 | 7 (35.0%) | 8.74 (1.83, 41.62) | 0.006 |  |
|  |  |  |  |  |  |
| **Facility Characteristics** |  |  |  |  |  |
| Academic/Research Program Facility Type | 999 | 262 (26.2%) | 2.89 (1.55, 5.38) | <.001 | <.001 |
| Geographic region |  |  |  |  | 0.019 |
| *East Coast* | 633 | 104 (16.4%) | 1.00 (ref) | - |  |
| *Central* | 388 | 75 (19.3%) | 1.87 (1.03, 3.40) | 0.041 |  |
| *Mountain* | 54 | 17 (31.5%) | 5.65 (1.47, 21.66) | 0.012 |  |
| *Pacific* | 941 | 117 (12.4%) | 1.03 (0.46, 2.29) | 0.951 |  |
| Patients/year quartiles |  |  |  |  | <.001 |
| *Q1* | 126 | 3 (2.4%) | 1.00 (ref) | - |  |
| *Q2* | 278 | 1 (0.4%) | 0.07 (0.00, 1.04) | 0.053 |  |
| *Q3* | 368 | 15 (4.1%) | 1.04 (0.29, 3.82) | 0.948 |  |
| *Q4* | 1244 | 294 (23.6%) | 3.88 (1.16, 12.97) | 0.027 |  |
